# Elucidating the Effect of Antenatal Corticosteroids in the Late Preterm Period

**DOI:** 10.1007/s13224-022-01664-5

**Published:** 2022-08-29

**Authors:** Rekha Upadhya, Sai Bhavana, Muralidhar V. Pai, Shweta Tahlan

**Affiliations:** 1 Department of Obstetrics and Gynaecology, Kasturba Medical College, Manipal Academy of Higher Education, Manipal, Karnataka 576104 India; 2 Department of Surgical Oncology, Homi Bhabha Cancer Hospital, Sangrur, India

**Keywords:** Antenatal corticosteroids, Late preterm, Respiratory morbidity, Respiratory distress syndrome, Neonatal intensive care unit (NICU), Hyperbilirubinemia, Phototherapy, Invasive ventilation

## Abstract

**Aim and Objective:**

To determine the efficacy of antenatal corticosteroids given in the late preterm period.

**Methodology:**

We conducted a retrospective case–control study on patients with singleton pregnancies who were at a risk of delivering in the late preterm period (34 weeks to 36 weeks 6 days). A total of 126 patients who had received antenatal corticosteroids (prenatal administration of either betamethasone or dexamethasone, minimum one dose) during the late preterm period were taken as cases, and 135 patients who had not received steroids antenatally due to various reasons, for example, who were clinically unstable, presented with active bleeding, non-reassuring foetal status that obligated an imminent delivery and those in active labour were included as controls. The various neonatal outcomes like APGAR score at one and five minutes, incidence of admission and duration of stay in neonatal intensive care unit (NICU), respiratory morbidity, requirement of assisted ventilation, intraventricular haemorrhage (IVH) necrotizing enterocolitis, transient tachypnea of the newborn, respiratory distress syndrome, use of surfactant, neonatal hypoglycaemia, hyperbilirubinemia requiring phototherapy, sepsis and neonatal mortality were compared between the two groups.

**Results:**

The baseline characteristics of both groups were comparable. There was a lower incidence of admissions to neonatal intensive care unit (NICU) (15% vs. 26%, *p* = 0.05), respiratory distress syndrome (5% vs. 13%, *p* = 0.04), requirement of invasive ventilation (0% vs. 4%, *p* = 0.04) and hyperbilirubinemia requiring phototherapy (24% vs. 39%, *p* = 0.02) in the babies of the group that received steroids compared to the control group. The rate of overall respiratory morbidity in the neonates was lowered after giving steroids (16% vs. 28%, *p* = 0.04). The incidence of neonatal necrotizing enterocolitis, hypoglycaemia, IVH, TTN, sepsis and mortality between the two groups was not significant (*p* > 0.05).

**Conclusion:**

Antenatal corticosteroids administered to patients between 34 and 36 weeks 6 days of gestation reduce respiratory morbidity, requirement of invasive ventilation, respiratory distress syndrome, hyperbilirubinemia requiring phototherapy and the incidence of NICU admissions in the newborns.

**Supplementary Information:**

The online version contains supplementary material available at 10.1007/s13224-022-01664-5.

## Introduction

Late preterm birth includes those deliveries that occur between 34 and 36 weeks 6 days. This subgroup constitute 75% of total preterm births (24–36 weeks, 6 days) [1] and 8% of deliveries [2]. The old school of thought was that late preterm infants were similar in physiological and metabolical maturity to the term infants. There is a general consensus now that they have a higher risk of morbidity and mortality as compared to those born at term [2–5]. These infants have a higher incidence of neonatal intensive care unit (NICU) admissions, RDS, TTN, pneumonia and low Apgar scores [6–8]. There is a higher incidence of periventricular leukomalacia (PVL), cerebral palsy and a poorer school performance in late preterm births [9].

Antenatal corticosteroids are strongly recommended in preterm deliveries prior to 34 weeks as their beneficial effects have been well established [10, 11]. Few studies done in the recent past have found the beneficial effects of steroids in the late preterm infants, especially in reducing respiratory morbidity [12].

Late prematurity is often associated with respiratory morbidity which requires additional oxygen and ventilatory support [12–14]. Transient tachypnoea of the newborn, respiratory distress syndrome, pneumonia and pulmonary hypertension are the most common respiratory disorders.

Corticosteroids easily cross the placenta [15]. They cause alveolar epithelial cell flattening, thinning of the septae, enhance the differentiation of pneumocytes, thus accelerating the foetal lung maturation. They also stimulate the type 2 pneumocytes and increase phospholipid synthesis, thus augmenting surfactant release [16]. They activate nitric oxide synthase in the endothelium [17], which increases the blood flow in the pulmonary blood vessels and thus improves the adaptation of the foetal lungs after birth. The epithelial sodium channels increase [18], and the fluid clears from the lumen of the alveoli to the interstitium; thus, ventilation-perfusion mismatch is prevented [19].

In this study, we aim to elucidate the beneficial effects of antenatal corticosteroids in the late preterm period.

## Methodology

We conducted a retrospective case–control study in a tertiary centre of South India conducted between January 2018 and June 2019. After obtaining clearance from the Institutional Ethics Committee, all the women with singleton pregnancies who had late preterm delivery between 34 and 36 weeks 6 days of gestation in our hospital in the past one and half years were enrolled in the study. Some of them received steroids, while some others did not due to various reasons. None of the patients who had late preterm delivery were given magnesium sulphate neuroprophylaxis as they were beyond 34 weeks of gestation. As per NICE guidelines, magnesium sulphate is given for neuroprotection of the baby in women ≤ 33 weeks 6 days. These women who had late preterm delivery were divided into 2 groups: (1) 126 cases - those who had received antenatal corticosteroids (prenatal administration of either betamethasone or dexamethasone, minimum one dose) during the late preterm period and (b) 135 controls - those who had not received steroids antenatally due to various reasons, for example, who were clinically unstable, presented with active bleeding, non-reassuring foetal status that obligated an imminent delivery and those in active labour. Patients with uncontrolled gestational or overt diabetes mellitus, preeclampsia, eclampsia, chorioamnionitis, chronic inflammatory diseases like systemic lupus erythematosus, rheumatoid arthritis, thromboembolic disorders, disseminated intravascular coagulation and mothers with structural or chromosomal anomalies in the foetuses were excluded from the study. Twenty-six patients among the cases and 35 among the controls were excluded as they met the above exclusion criteria. Thus a total of 100 cases and 100 controls were included in the study for analysis. The maternal characteristics like age, parity, use of assisted reproductive techniques, comorbidities, gestational age at delivery and the mode of delivery (vaginal, instrumental forceps or vacuum assisted, caesarean section) were recorded. The neonatal outcomes such as birth weight, NICU admission, duration of NICU stay, APGAR scores at birth, respiratory morbidity like TTN, meconium aspiration syndrome, RDS, requirement of invasive or non-invasive respiratory support (continuous positive airway pressure (CPAP) or intubation) and surfactant, hyperbilirubinemia requiring phototherapy, hypoglycaemia, intraventricular haemorrhage (IVH), necrotizing enterocolitis (NEC), sepsis and neonatal death were recorded by reviewing the medical records.

In both the groups, the categorical variables were expressed as percentages (%) and Chi-square test was used for comparison, while the continuous variables were expressed as mean ± standard deviation (SD) and Student t test was used for the comparison. SPSS version 21 was used for the statistical analysis. A *p* value of < 0.05 was considered to be statistically significant.

## Results

After applying the exclusion criteria, we had a total of 100 cases and 100 controls. The basic demographic and clinical characteristics are shown in Table 1. Both the groups were matched in terms of age, parity, use of artificial reproductive technology, comorbidities, gestational age at delivery and mode of delivery (*p* > 0.05).

**Table 1:** Maternal and clinical characteristics in both the groups

Maternal variables	Received steroids (cases) (*n* = 100) %	Did not receive steroids (controls) (*n* = 100) %	“*p*’’ value (< 0.05–significant)
Mean age ± SD (years)	27.8 ± 3.27	28 ± 3.77	0.689^#^
Primigravida	65	67	0.765*
ART	34	25	0.16*
Gestational diabetes	30	20	0.1*
Gestational hypertension	22	20	0.72*
*Gestational age at delivery (weeks)*			
Mean ± SD	34.6 ± 3.68	34.3 ± 3.54	0.557^#^
34 − 34 + 6	45	47	0.77*
35 − 35 + 6	43	37	0.38*
36 − 26 + 6	12	16	0.41*
Total	100	100	
*Mode of delivery*			
Vaginal	32	36	0.55*
Instrumental	2	2	1*
LSCS	66	62	0.556*
Total	100	100	

*SD* Standard deviation, *LSCS* Lower segment caesarean section, *ART* Assisted reproductive techniques

*Chi-square test, ^#^Student *t* test

As seen in Table 2, there was no significant difference in the birth weight, APGAR scores and the incidence of NEC, sepsis and neonatal deaths in the two groups (*p* > 0.05). The number of NICU admissions (26% vs. 15%, *p* = 0.05) and duration of NICU stay > 3 days (13% vs. 5%, *p* = 0.04) was significantly higher among the infants who did not receive steroids as compared to the cases. There was a lower incidence of hyperbilirubinemia requiring phototherapy in the neonates born to the group that received steroids as compared to the controls, and it was statistically significant (24% vs. 39%, *p* = 0.02). Neonatal outcomes like TTN, hypoglycaemia, sepsis and mortality were not statistically significant among the two groups. There were no cases of IVH and NEC in both the groups.

**Table 2:** Neonatal outcomes among the two groups

Neonatal outcomes	Received steroids (cases) (*n* = 100), %	Did not receive steroids (controls) (*n* = 100) %	“*p*’’ value < 0.05–significant
*Birth weight (kg)*			
Mean ± SD	2.1 ± 0.33	2.1 ± 0.38	1^#^
2.5 or more	18	19	0.855*
1.5- < 2.5	80	78	0.72*
< 1.5	2	3	0.65*
*APGAR score* < *9 at*			
1 min	4	10	0.09*
5 min	0	2	0.15*
NICU admissions	15	26	0.05*
NICU stay for > 3 days	5	13	0.04*
Hyperbilirubinemia requiring phototherapy	24	39	0.02*
IVH/NEC	0	0	–
Neonatal hypoglycaemia	18	13	0.32*
Sepsis	0	1	0.3*
Death	0	1	0.3*

*SD* Standard deviation

*Chi-square test, ^#^Student *t* test

Table 3 shows the respiratory morbidities in both the groups. The rates of RDS were significantly lower among the cases as compared to the controls (5% vs. 13%, *p* = 0.04). Among these late preterm infants, none developed severe RDS. Only 2 babies in the control group required exogenous surfactant. The rates of TTN and meconium aspiration syndrome (MAS) were similar (*p* > 0.05). The necessity for invasive ventilation was higher among the babies born to the controls (4% vs. 0%, *p* = 0.04), but the use of non-invasive ventilation was similar in both groups.

**Table 3:** Respiratory morbidities in both groups

Respiratory morbidities	Received steroids (cases) (*n* = 100), %	Did not receive steroids (controls) (*n* = 100) %	“*p*’’ value < 0.05–significant
TTN	12	14	0.67*
MAS	3	2	0.65*
RDS	5	13	0.04*
Mild	3	8	0.12*
Moderate	2	5	0.08*
Severe	0	0	
Surfactant use	0	2	0.15*
*Assisted ventilation*			
Non-invasive	6	12	0.14*
Invasive	0	4	0.04*

*Chi-square test, ^#^Student *t* test

The stratified analysis of incidence of respiratory morbidities in neonates born at different gestational ages is shown in Table 4. The overall incidence of respiratory morbidity was significantly higher in the control group (16% vs. 28%, *p* = 0.04), with no significant difference in the incidence among infants born > 35 weeks in both groups, while in those born at 34–34 weeks 6 days, there was a higher frequency of respiratory disorders in the control group (22% vs. 42%, *p* = 0.04). From this, we can deduce that the maximum beneficial effect of steroids is in the babies born < 35 weeks.

**Table 4:** Respiratory morbidities in neonates at different gestational ages

Variables	Respiratory morbidity *n* (%)	“*p*’’ value < 0.05–significant
All gestational ages		
Received steroids (*n* = 100)	16 (16)	0.04*
Did not receive (*n* = 100)	28 (28)	
34–34 + 6 weeks		
Received steroids (*n* = 45)	10 (22)	0.04*
Did not receive (*n* = 47)	20 (42)	
35–35 + 6 weeks		
Received steroids (*n* = 43)	6 (14)	0.54*
Did not receive (*n* = 37)	7 (19)	
36–36 + 6 weeks		
Received steroids (*n* = 12)	0 (0)	0.38*
Did not receive (*n* = 16)	1 (6.2)	

*Chi-square test, ^#^Student *t* test

## Discussion

Our retrospective case–control study showed that antenatal corticosteroids given prior to late preterm deliveries reduced respiratory morbidity, mainly the development of respiratory distress syndrome (RDS), the incidence of NICU admissions and the necessity of invasive ventilation in the neonates.

These findings were consistent with Gyamfi-Bannerman et al. who conducted a multicentric randomized control trial where there was a greater need for respiratory support within 72 h of birth in the placebo group (14.4%) as compared to the steroid group (11.6%) (*p* = 0.02, relative risk = 0.8). The steroid group had lower rates of TTN, severe respiratory complications and bronchopulmonary dysplasia. Based on this study, Society of Maternal–Fetal Medicine (SMFM) recommended that antenatal corticosteroids between 34 and 36 weeks 5 days should be administered only to women at the greatest risk of delivery like preterm labour with cervical changes (3 cm dilated or 75% effaced), preterm premature rupture of membranes or a planned late preterm delivery due to any obstetric indications like preeclampsia, intrauterine growth restriction, oligo- or anhydramnios. After the administration of steroids to the mother, the effect of endogenous corticosteroids in the foetus is accelerated which increases the surfactant production, thus preventing respiratory complications [16, 19, 20].

Our findings were in contrast to the results of the randomized clinical trial by Porto et al. in Brazil where they found similar rates of RDS (*p* = 0.54) and TTN (*p* = 0.77) in the placebo and steroid groups. The requirement of ventilatory support was 20% in both groups. Thus, they concluded that antenatal steroids between 34 and 36 weeks did not reduce respiratory disorders in newborns. They also deduced that babies in the steroids group required phototherapy less often for jaundice compared to the no-steroid ones (risk ratio = 0.63, 0.44–0.91). Our findings were also similar. This is probably due to acceleration of liver maturity by the corticosteroids [21].

A cohort study by Souter et al. in the Washington state highlighted the importance of vigilant selection of patients requiring steroid administration in the late preterm period. There was a higher prevalence of respiratory complications among newborns delivered at 34 and 35 weeks who were eligible for steroids as compared to the ineligible ones, while there was no difference among those delivered at 36 weeks. This finding was consistent with our study where we found a significantly higher rate of respiratory morbidities in the non-steroid group between 34 and 34 weeks 6 days than between 35 and 36 weeks 6 days.

The occurrence of neonatal hypoglycaemia was similar among both the groups in our study. This was comparable to the original trial of antenatal steroids where similar finds were noted [22]. However, some recent trials show that there is a higher occurrence of neonatal hypoglycaemia in the steroid group [12].

The American College of Obstetricians and Gynaecologists (ACOG) recommends that betamethasone can be given in those women between 34 and 36 weeks 6 days of gestation who are at a risk of preterm birth within 7 days and have not received a prior antenatal corticosteroid course [23].

Our study thus elucidates the beneficial effects of corticosteroid administration in the late preterm period.

Limitations: The patients who had received an earlier course of antenatal corticosteroids were not excluded in our study as the data were not available. The number of doses of the corticosteroids was not considered. The retrospective nature and the small sample size were some of the limitations of our study.

## Conclusion

Antenatal corticosteroids administered to patients between 34 and 36 weeks 6 days reduce respiratory morbidity, requirement of invasive ventilation, respiratory distress syndrome, hyperbilirubinemia requiring phototherapy and the incidence of NICU admissions in their newborns.

## Supplementary Information

Below is the link to the electronic supplementary material.

Supplementary file1 (PDF 568 KB)

Supplementary file2 (DOCX 18 KB)

Supplementary file3 (DOCX 14 KB)

## References

[1] Souter V, Kauffman E, Marshall A (2017). Assessing the potential impact of extending antenatal steroids to the late preterm period. Am J Obstet Gynecol.

[2] Young PC, Glasgow TS, Li X (2007). Mortality of late-preterm (near-term) newborns in Utah. Pediatrics.

[3] Kramer MS, Demissie K, Yang H (2000). The contribution of mild and moderate preterm birth to infant mortality. Fetal and infant health study group of the Canadian perinatal surveillance system. JAMA.

[4] Wang ML, Dorer DJ, Fleming MP (2004). Clinical outcomes of near-term infants. Pediatrics.

[5] Tomashek KM, Shapiro-Mendoza CK, Weiss J (2006). Early discharge among late preterm and term newborns and risk of neonatal morbidity. Semin Perinatol.

[6] McIntire DD, Leveno KJ (2008). Neonatal mortality and morbidity rates in late pre-term births compared with births at term. Obstet Gynecol.

[7] Yoder BA, Gordon MC, Barth WH (2008). Late-preterm birth: Does the changing ob-stetric paradigm alter the epidemiology of respiratory complications?. Obstet Gynecol.

[8] Hibbard JU, Wilkins I, Sun L (2010). Respiratory morbidity in late preterm births. JAMA.

[9] Kugelman A, Colin A (2013). Late preterm infants: near term but still in a critical developmental time period. Pediatrics.

[10] Crowley PA (1995). Antenatal corticosteroid therapy: a meta-analysis of the randomized trials, 1972 to 1994. Am J Obstet Gynecol.

[11] Roberts D, Dalziel S (2006). Antenatal corticosteroids for accelerating fetal lung maturation for women at risk of preterm birth. Cochrane Database Syst Rev.

[12] Gyamfi-Bannerman C, Thom EA, Blackwell SC (2016). Antenatal betamethasone for women at risk for late preterm delivery. N Engl J Med.

[13] Escobar GJ, Clark RH, Greene JD (2006). Short-term outcomes of infants born at 35 and 36 weeks’ gestation: we need to ask more questions. Semin Perinatol.

[14] Jain L, Eaton DC (2006). Physiology of fetal lung fluid clearance and the effect of labor. Semin Perinatol.

[15] Asztalos E (2012). Antenatal corticosteroids: a risk factor for the development and chronic disease. J Nutr Metab.

[16] Ballard P, Ballard R (1995). Scientific basis and therapeutic regimens for use of antenatal glucocorticoids. Am J Obstet Gynecol.

[17] Millage A, Latuga M, Aschner J (2016). Effect of perinatal glucocorticoids on vascular health and disease. Pediatr Res.

[18] Venkatesh VC, Katzberg HD (1997). Glucocorticoid regulation of epithelial sodium channel genes in human fetal lung. Am J Physiol.

[19] Jain L (1999). Alveolar fluid clearance in developing lungs and its role in neonatal transition. Clin Perinatol.

[20] Bonanno C, Wapner RJ (2009). Antenatal corticosteroid treatment: What’s happened since Drs Liggins and Howie?. Am J Obstet Gynecol.

[21] Porto AMF, Coutinho IC, Correia JB (2011). Effectiveness of antenatal corticosteroids in reducing respiratory disorders in late preterm infants: randomised clinical trial. BMJ.

[22] Liggins GC, Howie RN (1972). A controlled trial of antepartum glucocorticoid treatment for prevention of the respiratory distress syndrome in premature infants. Pediatrics.

[23] American College of Obstetricians and Gynecologists. Antenatal corticosteroid therapy for fetal maturation. Am J Obstet Gynecol. 2017; 130(2): e102. 10.1097/AOG.0000000000002237 28742678

