# Supplementary material for: Elucidating the Effect of Antenatal Corticosteroids in the Late Preterm Period
Source: J Obstet Gynaecol India. 2022 Aug 29;73(2):107–12. doi: 10.1007/s13224-022-01664-5 (PMC10105809; doi:10.1007/s13224-022-01664-5)
Supplement: Supplementary file 1 — Supplementary file1 (PDF 568 KB) [file 13224_2022_1664_MOESM1_ESM.pdf]

THE JOURNAL OF OBSTETRICS AND GYNECOLOGY OF INDIA
Copyright/Authorship/Disclosure Form

Springer

MANUSCRIPT ID NUMBER

Elucidating the effect of antenatal corticosteroids in the late preterm period

Article title (first few words)

First Author: Dr Rekha Upadhya

E-mail: rex2000@rediffmail.com

COPYRIGHT

The author(s) guarantee(s) that the manuscript will not be published elsewhere in any language without the consent of the copyright holder(s), that the rights of third parties will not be violated, and that the publisher will not be held legally responsible should there be any claims for compensation.

The Author of the Article as specified herein, hereby transfers copyright to The Federation of Obstetrics and Gynaecological Societies of India (respective to owner if other than Springer and for U.S. government employees: to the extent transferable) effective if and when the article is accepted for publication. The author warrants that his/her contribution is original and that he/she has full power to make this grant. The author signs for and accepts responsibility for releasing this material on behalf of any and all co-authors. The copyright transfer covers the exclusive right to reproduce and distribute the article, including reprints, translations, photographic reproductions, microform, electronic form (offline, online) or any other reproductions of similar nature. An author may self-archive an author-created version of his/her article on his/her own website. He/she may also deposit this version in any repository, provided it is not made publicly available until after 12 months of official publication. He/she may not use the publisher's PDF version which is posted on link.springer.com for the purpose of self-archiving or deposit. Furthermore, the author may only post his/her version provided acknowledgement is given to the original source of publication and a link is inserted to the published article on Springer's website. The link must be accompanied by the following text: "The original publication is available at link.springer.com".

Please use the appropriate DOI (digital object identifier) for the article (go to the Linking Options in the article, then to Open URL (Uniform Resource Locator) and use the link with the DOI). Articles disseminated via link.springer.com are indexed, abstracted, and referenced by many abstracting and information services, bibliographic networks, subscription agencies, library networks, and consortia.

AUTHORSHIP

I, the undersigned author(s), certify that:

- I have seen and approved the final version of the manuscript, and all subsequent versions.
- I have made substantial contributions to conception and design, or acquisition of data, or analysis and interpretation of data;
- I have drafted the article or revised it critically for important intellectual content.

I accept public responsibility for it, and believe it represents valid work. As an author of this article, I certify that none of the material in the manuscript has been previously published, nor is included in any other manuscript. I certify that this manuscript is not under consideration for publication elsewhere, nor has it been submitted or accepted in another publication in any form. The rights or interest in the manuscript have not been assigned to any third party.

Moreover, should the editor of The Journal of Obstetrics and Gynecology of India request the data upon which the manuscript is based, I shall produce it. I also certify that I have read and complied with the copyright information, as found on the journal home page website.

After submission of this agreement signed by all authors, changes of authorship or in the order of the authors listed will not be accepted by Springer.

THE JOURNAL OF OBSTETRICS AND GYNECOLOGY OF INDIA
Copyright/Authorship/Disclosure Form

Springer

MANUSCRIPT ID NUMBER

FINANCIAL DISCLOSURE/CONFLICT OF INTEREST

I certify that any financial interests such as employment, stock ownership, honoraria, paid expert testimony, as well as any personal relationships, academic competition, and intellectual passion which may inappropriately influence my actions, **have been included within my manuscript**. If none exist, the statement "Conflict of Interest: None" has been also been included.

All funding sources supporting the work and all institutional or corporate affiliations of mine are acknowledged in a footnote.

I have had full access to all the data in the study (if applicable) and thereby accept full responsibility for the integrity of the data and the accuracy of the data analysis.

By checking the box next to my signature I assert that there are no conflicts of interest (both personal and institutional) regarding specific financial interests that are relevant to the work conducted or reported in this manuscript.

☑ rekha
Author's signature
Rekha
Printed name and date

☑ sai bhavana
Author's signature
Sai Bhavana
Printed name and date

☑ Mv pai
Author's signature
Muralidhar V Pai
Printed name and date

☐
Author's signature

Printed name and date

☐
Author's signature

Printed name and date

☐
Author's signature

Printed name and date

☐
Author's signature

Printed name and date

☐
Author's signature

Printed name and date

Completed forms can be scanned and included as a pdf file during the online submission process as a supplemental file not for review.

Springer
http://www.springer.com/journal/13224

The Journal of Obstetrics and Gynecology of India
Editor-in-Chief: Balsarkar, G.
ISSN: 0971-9202 (print version)
ISSN: 0975-6434 (electronic version)
Journal no. 13224
