# Supplementary material for: Elucidating the Effect of Antenatal Corticosteroids in the Late Preterm Period
Source: J Obstet Gynaecol India. 2022 Aug 29;73(2):107–12. doi: 10.1007/s13224-022-01664-5 (PMC10105809; doi:10.1007/s13224-022-01664-5)
Supplement: Supplementary file 2 — Supplementary file2 (DOCX 18 KB) [file 13224_2022_1664_MOESM2_ESM.docx]

**References**

1. Souter V, Kauffman E, Marshall A, Katon J. Assessing the potential impact of extending antenatal steroids to the late preterm period. Am J Obstet Gynecol 2017;217. 461.e1–461.e7.

2. Young PC, Glasgow TS, Li X, Guest-Warnick G, Stoddard G. Mortality of late-preterm (near-term) newborns in Utah. Pediatrics 2007;119:e659-65.

3. Kramer MS, Demissie K, Yang H, Platt RW, Sauvé R, Liston R. The contribution of mild and moderate preterm birth to infant mortality. Fetal and Infant Health Study Group of the Canadian Perinatal Surveillance System. JAMA 2000;284:843-9.

4. Wang ML, Dorer DJ, Fleming MP, Catlin EA. Clinical outcomes of near- term infants. Pediatrics 2004;114:372-6.

5. Tomashek KM, Shapiro-Mendoza CK, Weiss J, Kotelchuck M, Barfield W, Evans S, et al. Early discharge among late preterm and term newborns and risk of neonatal morbidity. Semin Perinatol 2006;30:61-8.

6. McIntire DD, Leveno KJ. Neonatal mortality and morbidity rates in late pre- term births compared with births at term. Obstet Gynecol 2008;111:35-41.

7. Yoder BA, Gordon MC, Barth WH Jr. Late-preterm birth: does the changing ob- stetric paradigm alter the epidemiology of respiratory complications? Obstet Gynecol 2008;111:814-22.

8. Hibbard JU, Wilkins I, Sun L, et al. Respiratory morbidity in late preterm births. JAMA 2010;304:419-25.

9. Kugelman A, Colin A. Late preterm infants: near term but still in a critical developmental time period. Pediatrics 2013;132:741–51.

10. Crowley PA. Antenatal corticosteroid therapy: a meta-analysis of the randomized trials, 1972 to 1994. Am J Obstet Gynecol 1995;173:322-35.

11. Roberts D, Dalziel S. Antenatal corticosteroids for accelerating fetal lung maturation for women at risk of preterm birth. Cochrane Database Syst Rev 2006;3:CD004454.

12. C.Gyamfi-Bannerman, E.A. Thom, S.C. Blackwell, A.T.N. Tita, U.M. Reddy, G.R. Saade, D.J. Rouse,D.S. McKenna, E.A.S. Clark, J.M. Thorp, Jr., E.K. Chien, A.M. Peaceman, R.S. Gibbs, G.K. Swamy,M.E. Norton, B.M. Casey, S.N. Caritis, J.E. Tolosa, Y. Sorokin, J.P. VanDorsten, and L. Jain. Antenatal Betamethasone for Women at Risk for Late Preterm Delivery. N Engl J Med 2016; 374:1311-1320 DOI: 10.1056/NEJMoa1516783.

13. Escobar GJ, Clark RH, Greene JD. Short-term outcomes of infants born at 35 and 36 weeks’ gestation: we need to ask more questions. Semin Perinatol 2006;30:28-33.

14. Jain L, Eaton DC. Physiology of fetal lung fluid clearance and the effect of labor. Semin Perinatol 2006;30:34-43.

15. Asztalos E. Antenatal corticosteroids: a risk factor for the development and chronic disease. J Nutr Metab 2012;2012:930591.

16. Ballard P, Ballard R. Scientific basis and therapeutic regimens for use of antenatal glucocorticoids. Am J Obstet Gynecol 1995;173. 254–262.21.

17. Millage A, Latuga M, Aschner J. Effect of perinatal glucocorticoids on vascular health and disease. Pediatr Res 2016;81:4–10.

18. Venkatesh VC, Katzberg HD. Glucocorticoid regulation of epithelial sodium channel genes in human fetal lung. Am J Physiol 1997;273:L227–33.

19. Jain L. Alveolar fluid clearance in developing lungs and its role in neonatal transition. Clin Perinatol 1999;26:585–99.

20. Bonanno C, Wapner RJ. Antenatal corticosteroid treatment: what’s happened since Drs Liggins and Howie? Am J Obstet Gynecol 2009;200:448-57.

21. Ana Maria Feitosa Porto, Isabela Cristina Coutinho, Jailson Barros Correia, Melania Maria Ramos Amorim. Effectiveness of antenatal corticosteroids in reducing respiratory disorders in late preterm infants: randomised clinical trial. BMJ 2011;342:d1696, doi:10.1136/bmj.d1696

22. Liggins GC, Howie RN. A controlled trial of antepartum glucocorticoid treatment for prevention of the respiratory dis- tress syndrome in premature infants. Pediatrics 1972;50:515-25.

23. American College of Obstetricians and Gynecologists. Antenatal corticosteroid therapy for fetal maturation. Am J Obstet Gynecol August 2017;130(2):e102.
