# Supplementary material for: Elucidating the Effect of Antenatal Corticosteroids in the Late Preterm Period
Source: J Obstet Gynaecol India. 2022 Aug 29;73(2):107–12. doi: 10.1007/s13224-022-01664-5 (PMC10105809; doi:10.1007/s13224-022-01664-5)
Supplement: Supplementary file 3 — Supplementary file3 (DOCX 14 KB) [file 13224_2022_1664_MOESM3_ESM.docx]

**Abstract**

**Aim and objective:** To determine the efficacy of antenatal corticosteroids given in the late preterm period.

**Methodology:** We conducted a retrospective case control study on patients with singleton pregnancies who were at a risk of delivering in the late preterm period (34w to 36w 6day). 126 patients those who had received antenatal corticosteroids (prenatal administration of either betamethasone or dexamethasone, minimum of one dose) during the late preterm period were taken as cases and 135 patients those who had not received steroids antenatally due to various reasons, for example, who were clinically unstable, presented with active bleeding, nonreassuring fetal status that obligated an imminent delivery and in active labour were included as controls. After excluding those who met the exclusion criteria, a total of 100 cases and 100 controls were included in the study. The various maternal characteristics and neonatal outcomes such as APGAR score at one and five minutes, incidence of admission and duration of stay in neonatal intensive care unit(NICU) respiratory morbidity, requirement of assisted ventilation, intraventricular hemorrhage(IVH) necrotising enterocolitis(NEC), transient tachypnea of the newborn(TTN), respiratory distress syndrome(RDS), use of surfactant, neonatal hypoglycaemia, hyperbilirubinemia requiring phototherapy, sepsis and neonatal mortality were compared between the two groups.

**Results:** The baseline characteristics of both groups were comparable. There was a lower incidence of admissions to neonatal intensive care unit(NICU) (15% vs 26%, p=0.05), respiratory distress syndrome(5% vs 13%, p=0.04), requirement of invasive ventilation(0% vs 4%, p=0.04) and hyperbilirubinemia requiring phototherapy( 24% vs 39%, p=0.02) in the babies of the group that received steroids compared to the control group. The rates of overall respiratory morbidity in the neonates were lowered after giving steroids(16% vs 28%, p=0.04). The incidence of neonatal necrotising enterocolitis, hypoglycaemia, IVH, TTN, sepsis and mortality between the two groups was not significant(p>0.05).

**Conclusion:** Antenatal corticosteroids administered to patients between 34weeks to 36weeks 6days of gestation reduces respiratory morbidity, requirement of invasive ventilation, respiratory distress syndrome, hyperbilirubinemia requiring phototherapy and the incidence of NICU admissions in the newborns.
